# Effect of Blackcurrant Consumption on the Genitourinary System: A Literature Review

**DOI:** 10.7759/cureus.44181

**Published:** 2023-08-26

**Authors:** Meshari A Alzahrani, Faisal M Binnshwan, Khaled B Alsulaim, Osama A Mobeirek, Nasser M Albakran, Fahad A Albawardi, Abdulrahman I Almezaini, Yazeed K Alqahtani, Waleed Khalid Z Alghuyaythat, Ibrahim Abunohaiah, Raed AlAsmi, Raed Almannie

**Affiliations:** 1 Department of Urology, College of Medicine, Majmaah University, Al-Majmaah, SAU; 2 Medical School, College of Medicine, King Saud University, Riyadh, SAU; 3 Medical School, College of Medicine, Majmaah University, Al-Majmaah, SAU; 4 Department of Surgery, Division of Urology, Faculty of Medicine, King Saud University, King Saud University Medical City, Riyadh, SAU; 5 Department of Surgery, College of Medicine, Prince Sattam bin Abdulaziz University, Al‐Kharj, SAU

**Keywords:** antioxidant, infertility, genitourinary system, urolithiasis, blackcurrant

## Abstract

Both in vivo and in vitro studies have shown that functional plant-based food such as fruits, vegetables, and berries can enhance health, have preventive effects, and reduce the risk of several chronic diseases. This review discusses blackcurrant fruit usage in humans and experimental animals and its effect on the genitourinary system (GUS). This comprehensive review demonstrates that blackcurrants and their bioactive compounds possess medicinal and therapeutic properties related to the GUS. Emphasis in the literature has been placed on the bioavailability of the active blackcurrant components. Nonetheless, future clinical trials are needed to investigate and improve the bioavailability of blackcurrant phenolic compounds, such as anthocyanins, and to expand the evidence that active blackcurrant compounds can treat various genitourinary diseases.

## Introduction and background

**Figure 1:**
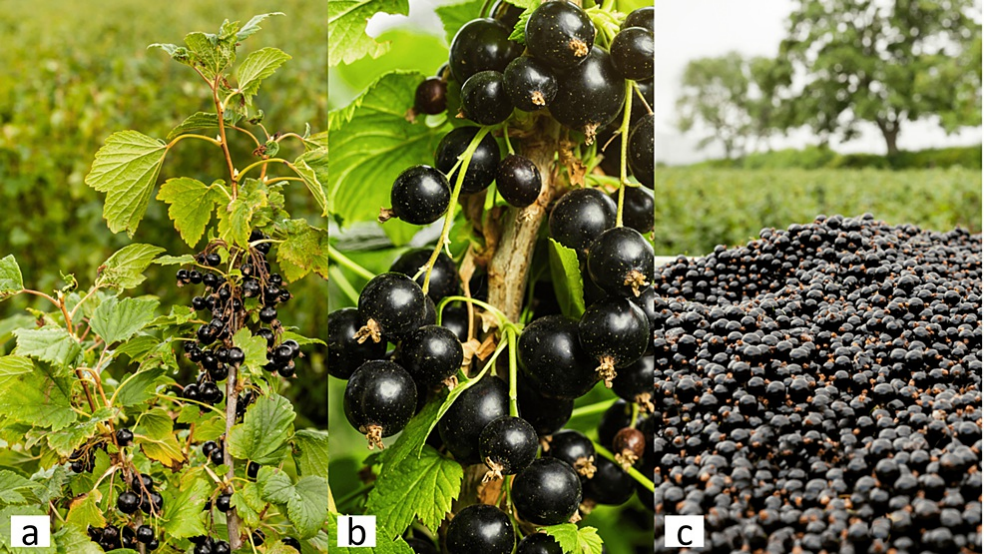
Blackcurrant shrub: (a): hairy gland-studded leaves of blackcurrant leafstalk, (b): spherical dark blackcurrant fruits, (c): harvested blackcurrants. [1]

The blackcurrant (BC) plant (Ribes nigrum L.) (Figure 1) is a small shrub with purple-black berries up to 12 mm in diameter and a persistent apical calyx with a shiny surface [1]. BC plants are upright aromatic deciduous bushes. They often lack spines and are composed of both short and long stems. The palmately vented, hairy, gland-studded leaves ranged from round to roughly triangular (their veins radiated from a common point near the leafstalk) (Figure 1A). The flowers are typically grouped and may be greenish, white, yellow, or reddish. In summer, mature shrubs can yield up to five kilograms of BC berries [2]. In summer, tiny glossy black fruits form on the stems. True berries are spherical and dark (Figure 1B). The harvest time lasts until mid-August when clusters of tiny, glossy black berries appear along the stems and can be plucked either manually or mechanically (Figure 1C) [3,4]. The cultivar is bred in Scotland, Poland, Lithuania, Latvia, Norway, and New Zealand to produce bushes with greater hardiness and disease resistance, as well as fruits of higher eating quality (Figure 1) [3,4].

BC fruits have higher levels of polyphenols than other berries, such as gooseberries, strawberries, red currants, raspberries, and white currants, in particular phenolic acid derivatives, anthocyanins, flavonols, condensed tannins (proanthocyanidins), flavan-3-ol (catechins), and hydrolysable tannins (ellagitanines and gallothanines) [2]. Thus, BC is seen as a fruit that can be utilized as a part of a healthy diet that is both affordable and profitable [5]. In the early 1900s, BC was declared illegal in the US as it was a vector for the white pine blister rust fungus, which had a detrimental effect on the logging industry. This restriction was lifted by several states in 2003 [2].

A recent narrative review published in 2021 reported that BC compounds can improve metabolic syndrome (MetS) risk factors, which may lead to a decreased risk of coronary heart disease (CHD) and type 2 diabetes mellitus (T2DM) [6]. Most studies on the impact of BC in medicine have examined its role in diabetes and cardiovascular health and its potent antioxidant properties. However, there is a lack of information regarding the therapeutic and protective roles of BC in genitourinary system (GUS) health. This review aimed to evaluate the current literature on the effects of BC on the GUS.

## Review

Metabolic effects of blackcurrant in animal studies

According to studies in rats, dietary blackcurrant seed oil (BCSO) improves lipid metabolism [7]. Male Wistar rats fed an adipogenic diet containing either canola oil (rapeseed oil) or BCSO for eight weeks showed a remarkable increase in body weight, altered plasma lipid profile, increased liver fat content, and increased plasma transaminase activity. An adipogenic diet also reduced short-chain fatty acid production and bacterial glycolysis in the distal intestine [7]. Consequently, the BCSO diet improved lipid metabolism by decreasing hepatic fat deposition, plasma triglyceride levels, and atherogenicity while increasing plasma HDL cholesterol levels. In rats fed an obesogenic diet containing BCSO, plasma high-density lipoprotein (HDL) cholesterol concentrations were the same as those in both diets, including canola oil, despite a significant increase in plasma transaminase activity [7]. In a menopausal animal model, BC anthocyanins reduced dyslipidemia. BC extract-treated rats had lower body weight, visceral fat weight, serum triglycerides, total cholesterol, and low-density lipoprotein (LDL) cholesterol [8].

Supplementation with BC leaf extract resulted in partially positive effects in dogs and cats with mild-to-moderate osteoarthritis [9]. After six weeks of feeding, rats fed anthocyanin-containing BC showed altered colon health biomarkers more effectively than rats without BC. In addition, the combination of dietary fiber (apple or broccoli) with BC is more effective in reducing body weight gain and food intake [10]. Studies in mice have demonstrated that BC extract suppresses M1-type macrophage polarization and reduces pro-inflammatory responses [11]. According to researchers, BC metabolites may not have a direct anti-inflammatory effect by altering macrophage phenotypes, but rather by inhibiting the production of obesity-associated inflammatory factors [11]. CCL11 has been linked to the early development of airway eosinophilia in allergic asthma. In a mouse model study, 10 milligrams per kilogram of oral New Zealand blackcurrant (NZBC) supplementation was found to be effective in reducing lung inflammation [12]. According to data from senescence-accelerated mice, anthocyanin-rich BC extract may be a useful food supplement or ingredient for the prevention of Alzheimer's disease [13]. According to an animal study, the early consumption of BC may protect against age-related bone loss. Tumor necrosis factor alpha (TNF-α) a proinflammatory cytokine that contributes to bone resorption, was significantly elevated in aged mice, but it was reduced by 43.3% with BC consumption [14]. Researchers have investigated whether phytoestrogenic activities could benefit the skin of menopausal women using ovariectomized rats and a normal human female skin fibroblast cell line (TIG113). They found that feeding ovariectomized rats with 3% BC extract daily for three months resulted in higher levels of collagen, elastin, and hyaluronic acid in the skin [15].

Effects of blackcurrant consumption on human health

Few studies have elucidated the effects of BC berries on human health (Table 1). Few randomized clinical trials have investigated the effects of BC on postprandial glycemia and insulinemia. These results suggest that BC can reduce postprandial glucose and insulin levels after a carbohydrate-rich meal [16-18]. Another study supported this finding and found that a product with fermented quinoa and 75 g of BC was able to lower postprandial glycemia and insulinemia, according to the results of a randomized crossover trial [19]. They also found that consuming both BC and blackcurrant purée (BCP) together led to lower glucose and insulin concentrations during the first 30 min, a more balanced decline during the first hour, and an improved glycemic profile [19]. In healthy men and women, a clinical trial showed that apple- and BC polyphenol-rich drinks reduced postprandial blood glucose concentrations, which may be partly related to the inhibition of intestinal glucose transport [16].

**Table 1:** Comparison of the most salient articles from the literature that directly speak to the issue of blackcurrant benefits reported from human studies. EIMD; exercise-induced muscle damage, CK; creatine kinase, LDL; low-density lipoprotein

Targeted body system	Potential Blackcurrant benefits	References
Endocrine system	Reduce postprandial glucose and insulin levels	[16-19]
Cardiovascular system	Changed cardiovascular responses, muscle oxygen saturation, muscular activity, femoral artery diameter, and enhanced vasodilation during sustained submaximal isometric exercise. Reduce acute endothelial dysfunction induced by smoking. Reduce the concentration of LDL cholesterol and increase plasma antioxidant capacity. Improved the levels of inflammatory markers in patients with peripheral arterial disease in combination with orange juice. Lowers central blood pressure and arterial stiffness.	[26-28,31,33]
Gastrointestinal system	Protects the gastrointestinal barrier. Improve the postprandial antioxidant status.	[34,36]
Immune system	Antioxidant and anti-inflammatory properties. Improve immunological reactivity.	[36,37]
Skin	Reduced the prevalence of chronic inflammatory skin diseases such as atopic dermatitis.	[38]
Musculoskeletal system	Influence exercise-induced physiological responses via higher oxidative capacities. Improved muscle oxygenation during and after contractions. Improved climbing endurance. Improved exercise-induced fat oxidation and burning. Reduce EIMD symptoms and facilitate faster muscle function recovery. following EIMD. Quicker return to baseline maximum voluntary contraction, reduced muscle pain at 24 and 48 h, and lower serum CK content. Reduced exercise-induced oxidative stress. Effective treatment for patients with active rheumatoid arthritis and synovitis.	[20-25,37,39]

BC contains anthocyanins, which may influence exercise-induced physiological responses. A study examined how BC affected muscle oxygenation and found that elite rock climbers who took BC extract for seven days had much higher oxidative capacities than the control group [20]. A similar study found that seven days of NZBC extract administration improved muscle oxygenation during and after contractions. However, neither arterial blood flow nor forearm endurance performance changed because of the clinical trial [21]. The NZBC extract facilitated not only the maintenance of total climbing time but also improved climbing endurance compared to a placebo, according to a clinical trial on sport climbing performance [22]. Daily use of anthocyanin-rich NZBC extract has been shown in clinical trials to be necessary for improving exercise-induced fat oxidation [23]. In physically active males, increased dietary anthocyanin intake was not related to body composition, but enhanced exercise-induced fat burning. The progressive accumulation and maintenance of anthocyanin-derived metabolites necessary to change the pathways for exercise-induced substrate oxidation appear to require daily anthocyanin consumption [23]. According to clinical trial reports, exercise-induced muscle damage (EIMD) symptoms are reduced by BC nectar [24], and BC extract has been reported to facilitate faster muscle function recovery following EIMD [25]. Ingestion of BC before and after eccentric exercise reduces muscle damage and inflammation. At 48 hours after exercise, oxygen radical absorption capacity was higher in the BC nectar group than in the control group [24]. After taking NZBC extract, there was a quicker return to baseline maximum voluntary contraction, reduced muscle pain at 24 and 48 h, and lower serum creatine kinase (CK) content at 96 h [25]. Furthermore, a clinical trial found that the consumption of NZBC extract consumption for seven days changed cardiovascular responses, muscle oxygen saturation, muscular activity, femoral artery diameter, and enhanced vasodilation during sustained submaximal isometric exercise in young, healthy men [26]. Together with this, changes in the cardiovascular system as a whole, a drop in muscle oxygen saturation and electromyography signal amplitude, and an increase in total hemoglobin occur. An increase in cardiac output and vasodilation suggests an increase in peripheral blood flow [26]. A clinical trial that used the flow-mediated dilatation (FMD) technique to evaluate endothelial function in patients who received a supplement containing 50 mg of BC anthocyanins concluded that BC anthocyanins could reduce acute endothelial dysfunction induced by smoking [27]. BC berries may also have a positive effect on cardiovascular health, as they are filled with anthocyanins, which can reduce the concentration of LDL cholesterol and increase plasma antioxidant capacity [28]. Another clinical trial revealed that a 20% (250 ml) BC juice drink had no appreciable effect on endothelial function biomarkers, lipid risk factors, or acute assessment of vascular reactivity [29]. They found that microbial metabolites of flavonoids were found in plasma after drinking BC juice, and anthocyanins were found in low concentrations in the urine; therefore, they concluded that drinking BC juice did not significantly affect vascular reactivity [29]. One study found that the NZBC extract did not improve cardiovascular function during rest and submaximal exercise in endurance-trained fed cyclists [30]. Furthermore, supplementation with orange and blackcurrant juices (500 ml/d) improved the levels of inflammatory markers in patients with peripheral arterial disease [31].

A recent clinical trial investigated the effects of acute supplementation with a patented BC beverage delivered alone or in combination with caffeine on repeated high-intensity cycling [32]. The results of this trial showed that the BC extract beverage, whether consumed alone or with caffeine, had no positive impact on physiological tests or cycling performance compared to a placebo. A recent clinical trial reported that short-term NZBC consumption lowers central blood pressure and arterial stiffness in elderly people, as reported in a recent clinical trial [33]. This trial suggests that NZBC can be used as an alternative to pharmaceutical medications. Therefore, anthocyanin-rich BC may be beneficial for maintaining or improving cardiovascular health.

During exertional heat stress, a clinical trial in human subjects examined the effects of consuming 600 mg/day of anthocyanin-rich BC extract for seven days on small intestine permeability, enterocyte damage, microbial translocation, and inflammation [34]. Researchers have found that BC extract protects the GI barrier. However, subclinical doses had no effect on microbial translocation or subsequent inflammatory processes.

Studies on human mononuclear blood cells (MNBC) using the comet assay to measure strand breaks (SB), endonuclease III (Endo III), and formamidopyrimidine DNA glycosylase (Fpg)-sensitive sites have been conducted to determine the effects of BC juice and BC anthocyanins on the steady-state level of oxidative DNA damage [35]. After three weeks of supplementation with daily doses ranging from 475 to 1000 ml/d, the baseline level of oxidative DNA damage was modest (less than 200 Fpg lesions per diploid cell), and the Fpg-sensitive sites increased throughout the intervention in the BC juice group [36]. According to this study, the subjects did not experience any reduction in oxidative DNA damage even with high dietary antioxidant intake. The results of this clinical study suggest possible antioxidant and anti-inflammatory properties of BC in vitro in cultured macrophages from human subjects. In this study, it was reported that the consumption of BC juice may improve the postprandial antioxidant status, as indicated by higher ascorbic acid levels and free radical scavenging ability in the plasma [36].

Clinical trials using human exercise and cellular models found that BC supplementation reduced exercise-induced oxidative stress, and that lipopolysaccharide (LPS)-stimulated production was blocked by antioxidants in BC extracts high in anthocyanins [37]. Additionally, BC promoted the early reduction of TNF-α and the anti-inflammatory characteristics of the differential temporal LPS-stimulated inflammatory response in THP-1 cells [37]. These results support the idea that consuming BC anthocyanins reduces oxidative stress and may if consumed at the right time and dose, supplement the ability of exercise to improve immunological reactivity to potential pathogens [37].

Supplementation with BCSO was well tolerated and momentarily reduced the prevalence of chronic inflammatory skin diseases such as atopic dermatitis [38]. The outcome of a 24-week clinical trial suggested that BCSO may be an effective treatment for patients with active rheumatoid arthritis and synovitis. Gammalinolenic acid (GLA) and alphalinolenic acid (ALA) were found to be abundant in BCSO [39].

Effect of blackcurrant on urinary tract health

Anthocyanidins are polyhydroxy and polymethoxy derivatives of 2-phenylbenzopyrylium (flavylium) cations. Anthocyanins are water-soluble glycosides and acylglycosides of anthocyanidins [40]. Flavan-3-ols, also known as proanthocyanidins (PAs), are both oligomeric and polymeric [41]. Although BC is not the only source of anthocyanins, they contain more anthocyanins than other berries and plants, with 476 mg/100 g of anthocyanins compared to 386 mg/100 g, 140 mg/100 g, and 122 mg/100 g for blueberries, cranberries, and cherries, respectively [42]. Additionally, certain New Zealand cultivars are exceptionally high in anthocyanins, with total anthocyanin concentrations varying between 346 and 850 mg/100 mL in NZBC juices [43,44], compared to 179-310 mg/100 mL in non-New Zealand cultivars [45]. The absorption and bioavailability of anthocyanins are complicated by their high susceptibility to heat and pH changes [46]. Anthocyanins may undergo several transformations before excretion, including enzymatic breakdown by liver microsomes and epithelial gut microbes [47]. The beneficial effects of anthocyanins on health could be attributed to both their absorbed intact structures and the bioactivity of their metabolites [48]. The highest concentrations of cyanidin-3-O-rutinoside (Cy3rut, antirrhinin) and delphinidin-3-O-rutinoside (Del3rut, tulipanin) were observed in BC [6]. The bioavailability of these anthocyanins and their degradation products, gallic acid and protocatechuic acid, was investigated by Röhrig et al. [49]. They discovered that plasma and urine concentrations peaked two hours after ingestion, with recoveries of 0.040% and 0.048% for Del3rut and Cy3rut, respectively [49]. After consuming BC, this study discovered significant amounts of bioactive degradation products in the plasma and urine, indicating a plethora of pathways for the breakdown of anthocyanins and their degradation products including Del3ruta and Cy3rut [49]. Howell et al. [50] identified proanthocyanidins in cranberries as compounds that are responsible for the inhibition of Escherichia coli adherence to uroepithelial cells. Bodel et al. [51] and Kahn et al. [52] observed acidifying urine after consumption of large amounts of cranberry juice. Following the administration of 100 cc of BC juice, Klingeberg et al. reported a modest drop in urine pH [53].

The role of BC in urinary tract infection (UTI) prevention and prophylaxis has not yet been studied, whereas the role of cranberry products as a preventive agent against UTI has been extensively demonstrated by six meta-analyses and many systematic reviews encompassing 82 clinical studies [54-59]. Uncertainties surrounding the effectiveness of cranberry products remain. A Cochrane review and meta-analysis discovered that cranberry products did not significantly lower the incidence of symptomatic UTI in women with recurrent UTIs compared with placebo, water, or no treatment [55]. According to five subsequent meta-analyses, cranberry-containing products may prevent UTIs in some patient populations [54,56-59]. The clinical and methodological heterogeneity of the included studies could be a contributing factor to the disparate results across meta-analyses [60].

According to the most recent 2023 European Association of Urology (EAU) panel consensus regarding prophylaxis with cranberries, clinicians may recommend cranberries for recurrent UTI prevention in women who are aware of the weak evidence base because of their favorable benefit-to-harm ratio. However, there is no clear clinical evidence regarding the optimal treatment dose and duration of treatment [61].

However, the effect of BC on kidney stone risk factors is poorly understood. Some evidence suggests BC prevents kidney stone formation (Table 2). A study was conducted to determine the effects of BC consumption on the risk factors of stone formation in the kidneys, and the results indicated that BC juice could support the treatment of uric acid stones because of its alkalizing properties [62]. In this study, BC juice increased urinary pH and citric acid excretion, which may potentially support the treatment and metaphylaxis of uric acid stones owing to its alkalizing impact [62]. In a previous study by Kessler et al. [62], results were obtained in healthy subjects, and further research is needed to evaluate the effects of BC juice on urine parameters in patients with stones. In addition, oxalic acid excretion significantly increased after exposure to BC juice. The BC juice's high concentration of oxalic acid (204 μmol/l) and, in particular, ascorbic acid (177 mg/100 g), which is intermediately converted to oxalic acid and excreted in the urine, contributes to the rise in oxalic acid excretion [62]. In addition, BC increases urinary citric acid, a well-known calcium oxalate (CaOx) inhibitor, by reducing the amount of calcium binding to oxalate to create CaOx stones; thus, BC is considered protective against CaOx stone formation in the urinary tract [63]. The alkalinizing action of BC juice and the subsequent increase in citric acid excretion are explained by the citric acid content (2.88 g/100 g), the daily intake of approximately 9 g, and its conversion to bicarbonate [64]. The role of BC in the prevention of urolithiasis is promising and further clinical trials are warranted.

Effects of blackcurrant on reproductive health

To the best of our knowledge, no human studies have shown how BC affects reproductive health. Some evidence suggests BC had a possible positive effect on reproductive health among animal studies (Table 2). However, one study was conducted in rats after exposure to oxidative stress due to exposure to diesel exhaust (DEE) fumes produced by the combustion of first- and second-generation biofuels. In this study, dietary interventions with BC pomace (20 g/kg feed) were administered to exposed rats. They observed a decrease in antioxidant defense systems, such as glutathione (GSH), higher levels of oxidized glutathione (GSSG), and a lower GSH/GSSG ratio [65]. They found that BC could reduce these negative effects by decreasing the oxidative stress markers thiobarbituric acid-reactive substances (TBARS), lipid hydroperoxides (LOOHs), 25-dihydroxycholesterols (25(OH)2Ch), and 7-ketocholesterol (7-KCh) [65]. In an interventional animal laboratory study, dietary intervention with BC pomace (20 g/kg feed) protected rats from testicular oxidative stress induced by exposure to biodiesel exhaust [65].

**Table 2:** Potential benefits of blackcurrant consumption on genitourinary health. BC; Blackcurrant, CaOx; Calcium Oxalate.

Blackcurrant consumption benefits	Type of studies	Reported outcome	References
Urolithiasis Prevention	Human studies	Increased urinary pH. Increased citric acid excretion. Increased oxalic acid excretion. BC prevents kidney stone formation. Protective against CaOx stone formation in the urinary tract. Treatment and metaphylaxis of uric acid stones due to BC alkalizing properties.	[62-64]
Reproductive Health	Animal studies	Antioxidant defense systems. Decreasing the oxidative stress markers. Protected rats from testicular oxidative stress induced by exposure to biodiesel exhaust.	[65]
Male Sexual Health	Human study	BC consumption with exercise can reduce the risk of erectile dysfunction.	[73]
Women Health	Human study	Reduced the risk of postmenopausal bone loss, possibly by improving bone formation.	[82]

The production and increase in abnormal sperm, as well as the reduction in sperm count, sperm transformation, and DNA fragmentation, are all significantly influenced by oxidative stress caused by free radicals. Consequently, infertility is caused by alterations to sperm DNA [66]. BC is a major source of vitamin C (l-ascorbic acid) [2]. The amount of vitamin C in BC has been examined in numerous studies and has been found to range from 70 to 280 mg per 100 g of fresh fruit. Hence, it is a primary source of this essential vitamin that is responsible for BC potent antioxidant benefits [2,67-69]. Vitamin C protects spermatogenesis in the male reproductive system. It also plays a significant role in semen integrity and fertility in humans and animals, boosts testosterone levels, and prevents sperm agglutination [70].

The BC contains numerous flavonoids [2]. According to a growing body of research, an increase in dietary flavonoid consumption is associated with improvements in endothelial function and blood pressure [71,72], indicating that flavonoids may enhance erectile function (EF) more than other dietary components [73].

Many plant-based foods and beverages, such as fruit, vegetables, tea, and herbs, contain flavonoids. Flavonoids have anti-inflammatory properties, inhibit the oxidation of low-density lipoprotein (LDL) and endothelial Nicotinamide adenine dinucleotide phosphate (NADPH) oxidase, regulate the activity of endothelial nitric oxide (NO) synthase, and increase NO status [71,74-78]. Combining flavonoid-rich foods such as BC with exercise can reduce the risk of erectile dysfunction (ED). Data from an observational study suggests that higher routine consumption of particular foods high in flavonoids is linked to a lower incidence of ED. The flavonoid polymer subclass contributed most to total flavonoid intake (mean intake 207 mg/d; range:68-442 mg/d), whereas intake ranging from 3.3 to 35.9 mg/d for anthocyanin and 13.6 to 102.5 mg/d for flavanones [73]. They found that individuals with a high intake of anthocyanins and flavanones, and high levels of physical activity, had a 21% lower risk of ED than those with a low intake of anthocyanins and flavanones and poor levels of physical activity [73].

Dyslipidemia is more common in postmenopausal women with low estrogen levels [79]. BC anthocyanins, alternatively, may dampen this response via phytoestrogen signaling in estrogen receptors [80,81]. Furthermore, a clinical study reported that the daily consumption of 784 mg of BC powder over six months reduced the risk of postmenopausal bone loss, possibly by improving bone formation. In addition, there was a substantial increase in the level of the bone formation indicator P1NP, which is the serum amino-terminal propeptide of type 1 procollagen [82].

Based on the previous literature, we believe that BC has a possible positive effect on male and female reproductive health. Further clinical studies are warranted to corroborate the relationship between BC and human reproductive health.

Discussion

Blackcurrants are known for their rich content of vitamin C, antioxidants, and flavonoids [2]. According to our review, These natural compounds have been suggested to benefit the health of the GUS. For example, BC extract has been reported to have anti-inflammatory effects [36,37], which can help to boost the immune system [83]. A review study reported infusions of BC leaves were used to speed up the process of elimination of toxins from the body and to regulate kidney function. These extracts were employed as diaphoretic and diuretic agents, as well as to treat inflammatory illnesses such as rheumatoid arthritis [84].

BC may reduce inflammation associated with UTIs and other conditions affecting the GUS. Additionally, some research suggests that BC has alkalizing effect by increased citric acid excretion, which may promote urinary flow and help prevent UTIs [62]. Although current research on the direct effect of BC on UTIs is limited, some studies propose that high vitamin C intake might help prevent or alleviate UTIs [85]. Further research is imperative to ascertain BC's efficacy in treating or preventing UTIs.

The body of research specifically investigating the impact of BC on urolithiasis is limited. However, some studies suggest that BC may have a beneficial effect on kidney health and may help prevent the formation of urinary stones due to its high content of antioxidants, which may reduce inflammation and oxidative stress in the kidneys as well as BC have alkalinizing action that increases in increased urinary pH and citric acid excretion which protects against urolithiasis formation [62-64]. Nevertheless, more research is needed to determine the specific effects of BC on urolithiasis.

There is limited scientific evidence available on the effects of BC on men’s and women’s reproductive health. As a dietary supplement, BC is generally considered safe for most people when taken in recommended dosages. There is some evidence to suggest that BC may have a positive impact on fertility, particularly in men. BC is high in antioxidants such as anthocyanins, which have been linked to improved sperm quality and motility [2,65-69]. However, more research is needed to confirm these findings and determine the optimal amount of BC needed to have a significant effect on fertility.

There is limited scientific evidence to suggest that consuming BC directly affects EF in humans. However, some studies suggest that blackcurrants and their extracts may have potential benefits in improving blood flow and reducing oxidative stress [71-78]. Both of these factors can play a role in EF. Nevertheless, more research is needed to determine the potential therapeutic effects of blackcurrants on EF in humans.

Limitations

To the best of our knowledge, this is the first review of BC and its effects on the GUS. The lack of homogeneity among the studies made it difficult to conduct a systematic review and meta-analysis. Although reviews have been criticized for their lack of rigor and synthesis, they can be larger in scope than systematic reviews. It is important to mention that available literature lacks evidence of interaction with another fruit or medication as well as on the cost of BC.

Recommendation and future direction

Studies on chronic BC supplementation in humans may validate the encouraging findings from animal models and human trials. Further studies are required to understand the processes underlying the potentially beneficial effects of BC on human health. To clearly define the effectiveness of BC nutrient qualities, the necessary dose of BC, duration of supplementation, and results on the human GUS must be determined. Moreover, more mechanistic investigations and RCTs are required to supplement the current lack of evidence.

## Conclusions

This review aimed to collect and highlight scientific evidence regarding the role of BC consumption in the prevention of GUS diseases. Present evidence indicates that BC positively influences the management of several MetS risk factors such as dyslipidemia, hyperglycemia, and hypertension, demonstrating anti-inflammatory properties as well. In addition, a growing body of evidence supports the effects of BC on GUS health. BC has the potential to increase citric acid content in the urine, thereby promoting alkalinization. Therefore, BC is believed to protect against urolithiasis, particularly CaOx and uric acid stones. However, the effectiveness of BC in preventing urinary tract infections is still unknown. BC is a potentially effective antioxidant because it contains high amounts of anthocyanins, a subclass of flavonoids, and is an important source of vitamin C (l-ascorbic acid), which increases testosterone levels, promotes spermatogenesis, and reduces DNA fragmentation. Furthermore, BC improves endothelial function, which may reduce the risk of erectile dysfunction. Due to limited evidence, quality, and robustness of available literature, further clinical studies on the beneficial effects of BC on genitourinary health are required.
